# The predictive value of deep learning-based cardiac ultrasound flow imaging for hypertrophic cardiomyopathy complicating arrhythmias

**DOI:** 10.1186/s40001-022-00975-7

**Published:** 2023-01-19

**Authors:** Haotang Wu, Zhiyong Huang, Juanjuan Liu, Jiancheng Dai, Yong Zhao, Weiquan Luo

**Affiliations:** 1 Department of Ultrasound, Zhongshan Hospital of Traditional Chinese Medicine, No. 3, Kangxin Road, West District, Zhongshan, 528400 China; 2 Department of Internal Medicine-Cardiovascular, Zhongshan Hospital of Traditional Chinese Medicine, Zhongshan, 528400 China

**Keywords:** Deep learning, Cardiac ultrasound flow imaging, Hypertrophic cardiomyopathy, Arrhythmias, Predictive value

## Abstract

**Objective:**

To investigate the predictive value of deep learning-based cardiac ultrasound flow imaging for hypertrophic cardiomyopathy (HCM) complicated by arrhythmias.

**Methods:**

The clinical data of 158 patients with hypertrophic cardiomyopathy were retrospectively collected from July 2019 to December 2021, and additionally divided into training group 106 cases, validation group 26 cases and test group 26 cases according to the ratio of 4:1:1, and divided into concurrent and non-concurrent groups according to whether they were complicated by arrhythmia or not, respectively. General data of patients (age, gender, BMI, systolic blood pressure, diastolic blood pressure, HR) were collected, a deep learning model for cardiac ultrasound flow imaging was established, and image data, LVEF, LAVI, *E*/*e*', vortex area change rate, circulation intensity change rate, mean blood flow velocity, and mean EL value were extracted.

**Results:**

The differences in general data (age, gender, BMI, systolic blood pressure, diastolic blood pressure, HR) between the three groups were not statistically significant, *P* > 0.05. The differences in age, gender, BMI, systolic blood pressure, diastolic blood pressure, HR between the patients in the concurrent and non-concurrent groups in the training group were not statistically significant, *P* > 0.05.

**Conclusions:**

Deep learning-based cardiac ultrasound flow imaging can identify cardiac ultrasound images more accurately and has a high predictive value for arrhythmias complicating hypertrophic cardiomyopathy, and vortex area change rate, circulation intensity change rate, mean flow velocity, mean EL, LAVI, and *E*/*e*' are all risk factors for arrhythmias complicating hypertrophic cardiomyopathy.

## Introduction

The global prevalence of hypertrophic cardiomyopathy is 1:500, with an estimated 1.1–2.8 million patients in China [1]. The incidence of hypertrophic cardiomyopathy and death rates are still on the rise worldwide [2]. Hypertrophic cardiomyopathy is a chronic progressive disease caused by excessive myocardial contraction and impaired blood filling of the left ventricle and is a relatively rare cardiovascular disease [3]. Because patients may not have obvious symptoms and have symptoms similar to other diseases, only a minority of cases are clinically diagnosed and it is estimated that about 80–90% of patients are undiagnosed [4]. There is no cure for hypertrophic cardiomyopathy and the prognosis for most patients is poor [5]. Once patients with hypertrophic cardiomyopathy become symptomatic, the disease progressively worsens and in later stages can be combined with cardiovascular disease such as heart failure, arrhythmias and stroke, and is the leading cause of death in older patients with hypertrophic cardiomyopathy [6, 7]. Patients with dyspnoea, chest pain, palpitations, fatigue and syncope should seek immediate medical attention from a cardiology department. Further diagnosis and active treatment through ultrasound testing can slow the progression of the disease, prevent sudden death and heart failure, and improve quality of life [8, 9]. Its potential to cause arrhythmias, which can be serious enough to cause sudden death [10], also puts patients with HCM under great psychological stress and seriously affects their quality of life.

Doppler ultrasound flow imaging is a simple, convenient and non-invasive technique that is now widely used [11]. Conventional cardiac ultrasound images do not reflect the variability of LV function and haemodynamics, limiting their use in clinical practice, whereas ultrasound flow imaging can provide complex and realistic information on cardiac blood flow dynamics at a lower cost [12]. It has been shown that ultrasound flow imaging in patients with HCM can clearly display their left ventricular haemodynamic parameters and identify their clinical phenotype, allowing for more accurate prediction of HCM [13]. Cardiac ultrasound flow imaging is a common tool for the detection of hypertrophic cardiomyopathy, but there may be diagnostic inaccuracies in manual judgement of its imaging [14], with the development of artificial intelligence, represented by deep learning With the development of artificial intelligence, artificial intelligence technology represented by deep learning has become an auxiliary tool for various imaging techniques, which takes raw image data as the basis, learns higher-order features of the image through multilayer neural networks, and the network automatically extracts the features for reorganisation and attribute categorisation and finally uses them to identify feature images, thus solving practical clinical problems [15, 16]. This study investigates the predictive value of deep learning-based cardiac ultrasound blood flow imaging models for the prevention of arrhythmias in patients with hypertrophic cardiomyopathy.

## Material and methods

### General information

The clinical data of 158 patients with hypertrophic cardiomyopathy were retrospectively collected from July 2019 to December 2021 and additionally divided into training group 1 06 cases, validation group 26 cases, and test group 26 cases according to the ratio of 4:1:1, and divided into concurrent and non-concurrent groups according to whether they were complicated by arrhythmias or not, respectively.

This study was conducted with the approval of our ethics committee.

Inclusion criteria: met diagnostic criteria for hypertrophic cardiomyopathy [17]; left ventricular posterior wall thickness or septal thickness ≥ 13 mm and left ventricular outflow tract pressure ≥ 20 mmHg.

Exclusion criteria: combination of other serious cardiovascular disease; previous history of cardiac disease; combination of serious cardiovascular disease; combination of serious arrhythmias; incomplete data.

### Methodology

#### Collection of information

General patient information is collected through the electronic medical record, which includes age, gender, BMI, systolic blood pressure, diastolic blood pressure, HR, in addition to extracting data using a deep learning model in the patient's cardiac ultrasound flow imaging, which includes LVEF, LAVI, E/e', rate of change of vortex area, rate of change of circulatory intensity, mean blood flow velocity, mean EL value.

#### Cardiac ultrasound flow imaging tests

##### Test equipment

The instrument is a Hitachi Aloka ProsoundF75 colour Doppler ultrasound diagnostic device with a 15–5 MHz probe and image processing using the DAS-RS1 ultrasound VFM image post-processing workstation. Testing is performed by a physician with more than 6 years of clinical experience in our department.

##### Test methods

**Fig. 1:**
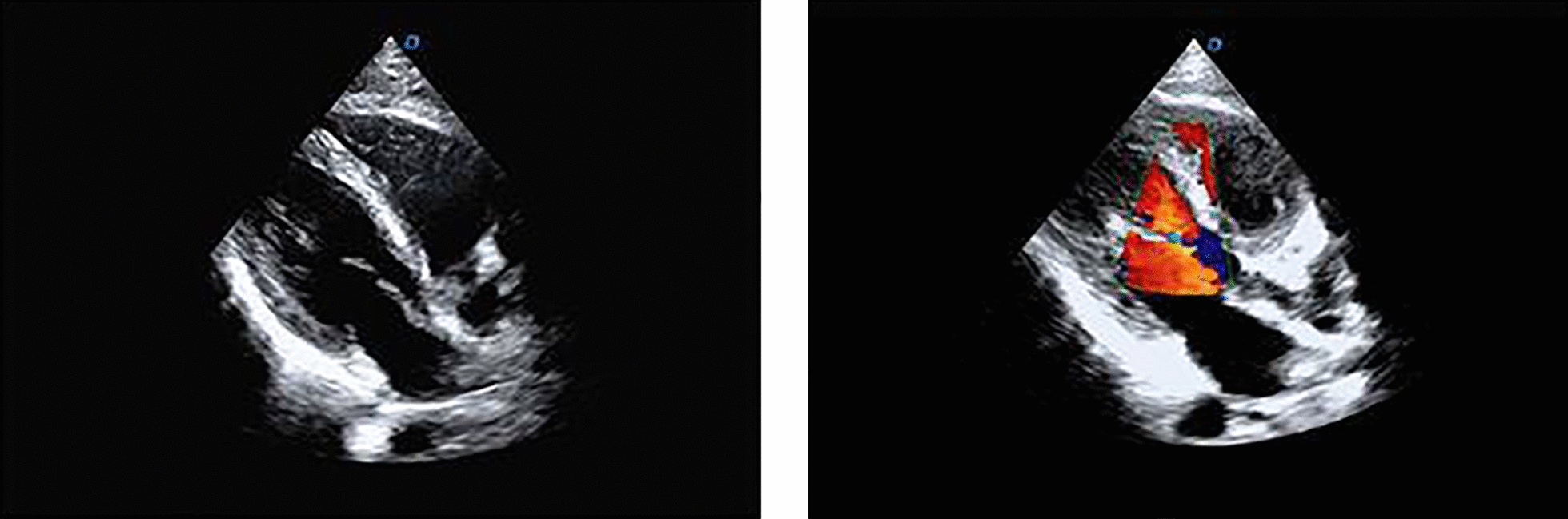
Ultrasound flow imaging of the heart

The patient was placed in the left lateral recumbent position at rest, the probe was placed on the patient's left ventricular apex, the imaging condition was adjusted to VFM, standard apical 2-chamber, 3-chamber and 4-chamber combined 3 complete cardiac circulation imaging was acquired and completely surrounded, and the imaging frame rate was adjusted to greater than 18 Hz. Using a dual Doppler simultaneous sampling technique, pulse Doppler or Doppler (PW or TDI), with PW and TDI set on the diastolic mitral orifice and the lateral wall of the left ventricular annulus, respectively, to capture synchronous motion rates, and PW or PW-type on a three-chamber standard three-chamber view, with sampling volumes set on the mitral and aortic orifices to capture synchronous flow spectra and measure IVC times. Ultrasound flow imaging of the heart is shown in Fig. 1.

##### Imaging analysis

(1) Echocardiographic images of apical four-chamber and two-chamber views were acquired, and the length of the biplane area was measured and calculated using the Simpson formula. (2) The acquired ultrasound images were input into the DAS-RS1 ultrasound workstation for offline analysis of VFM parameters: ① vortex area—concentric, closed flow line, outermost ring area is the vortex area, and the rate of change of vortex area was taken for early IVC versus late IVC; ② circulation intensity—vortex was measured through the centre of the spiral and a sampling line perpendicular to the ultrasound beam, and the circulation intensity was taken for early IVC versus late IVC rate of change; (iii) blood flow velocity—an anatomical flow line from the top to the bottom was set and the blood flow velocity gradient was applied from the tip of the heart to the left ventricular outlet area; the average of the three cross-sections was obtained by calculating the blood flow velocity for 2, 3 and 4 chambers, and the average blood flow velocity for the top + middle + bottom was taken; (iv) EL values—were derived using the VFM method, and the average EL for the top + middle + bottom was taken.

#### Image post-processing

Ultrasound image analysis was performed by CVI42 (Circle Cardiovascular Imaging Inc., Calgary, Alberta, Canada) software. Two specialists with more than 6 years of clinical experience performed the analysis of the basal, mid- and apical segments, and if the results differed between the two physicians, another senior physician (more than 10 years) made the determination. The ITK-SNAP software (version: 3.6.0) was used to segment the HCM images. Short-axis images of the basal, intermediate and apical segments were entered into the software separately and plotted between the ventricle and epicardium. Two cardiovascular imaging specialists with more than 6 years of clinical experience performed the image segmentation independently.

#### Deep learning model training

The deep learning model is SE -ResNext- 50, the overall deep learning training is done using the Pychar m compiler (https://www.jetbrains.com/pycharm/), the language used is Python 3.7, the deep learning framework is pytorch 1.0.4 (ht:/pytorch.or/), and the GPU model is NVIDIATESLA V100.

The image data of all HCM patients are first transformed into multiple 2D matrix forms and input into this network (2D input matrix size: 80 × 80), which is feature extracted using 32 parallel stacked residual blocks and the 32 features are combined. Due to the dependency between the convolution channels, this paper proposes a SE mechanism where the input image is assumed to be H*W*C (H and W represent the width and width of the input image, respectively, and C represents the number of channels in the input image), towards which it is expanded to 1*1*C by global pooling, an operation also known as the Squeeze operation. This is followed by two full convolution (FC) layers, which model the correlation between channels through the bottleneck structure and are activated between FC layers through ReLU, thus improving the sparsity of the network and better fitting the complex correlation between channels, while also reducing the number of parameters and the number of operations. In addition, a normalised weighting from 0 to 1 is obtained using Sigmoid and each channel is weighted using the scale operation, a process of activity assignment also known as the Excitation operation. Finally, the network input is combined with the SE mechanism to process the image and feed it into the next step of the network.

**Fig. 2:**
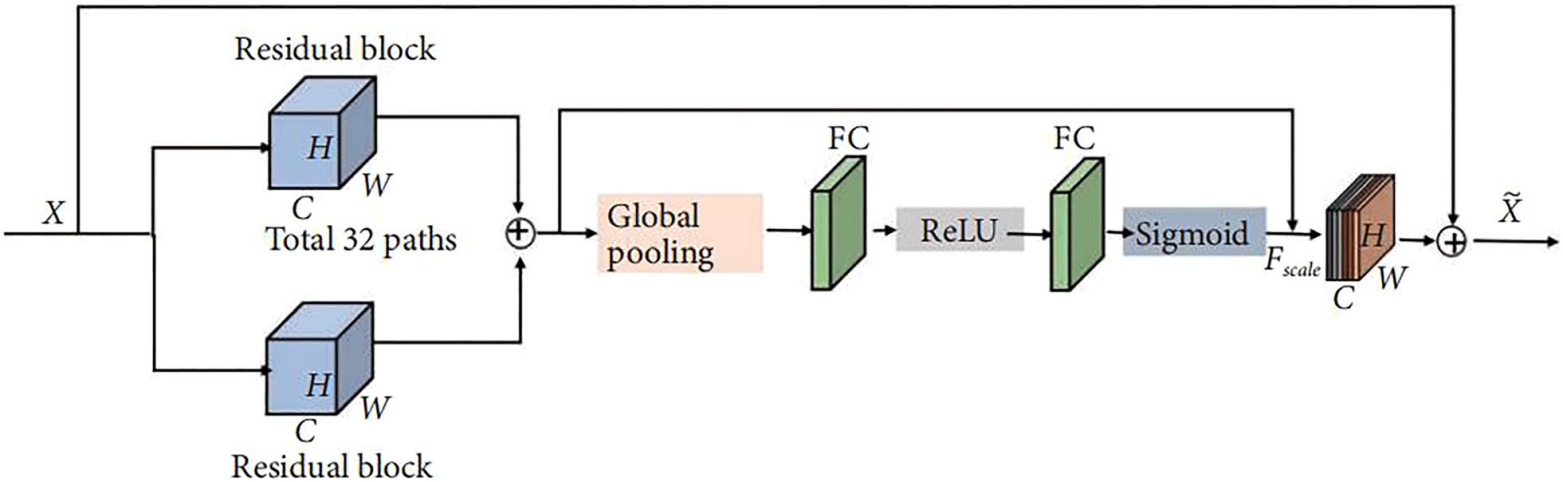
Workflow diagram of the SE-ResNext- 50 model

In response to the gradient diffusion problem prevalent in the inverse transmission of deep networks, the BN layer is introduced in this study by normalising the output data after the convolution of each layer. This method can effectively avoid the activation function ReLU from entering the non-linear saturation region, speed up the convergence of the network and prevent overfitting, thus enhancing the generalisation capability of the network (see Fig. 2).

Epoch (period) within the training group, validation group, and test group once each, thus facilitating control of the quality of the deep learning network training and obtaining the optimal test results. This study deals with a binary classification problem, so the predicted probability of 0–1 should be output in the last layer of this network, using One-Hot coding to obtain a 0 or 1 result, and calculating the CrossEntropyLoss with the original data labels, using this Loss metric to determine how close the actual output is to the desired output. Since the stochastic gradient algorithm may not follow the correct direction at each update and is prone to oscillate at the optimal solution and stop at the local optimum, this study uses the stochastic gradient descent algorithm SGDM with momentum, which accelerates if the update direction at this moment in the gradient descent is the same as the update direction at the previous moment, and decelerates if the opposite is true, and can accelerate the Loss descent and converge to the global optimum. At the same time, the learning rate is decayed by a factor of 0.1 if the 10-round Loss descent is not significant.

### Observation indicators

To compare general patient data, factors influencing hypertrophic cardiomyopathy complicating arrhythmias detected by cardiac ultrasound flow imaging based on deep learning models and the predictive value of the models.

### Statistical methods

SPSS26. 00 software was used to analyse the data in this study and the measures collected (age, BMI, systolic blood pressure, diastolic blood pressure, HR, rate of change of vortex area, rate of change of circulatory intensity, mean blood flow velocity, mean EL, LVEF, LAVI, *E*/*e*') were tested for normality by the Shapiro–Wilk method, *P* > 0.05 for normally distributed data expressed as (mean ± standard deviation), t-test, *P* < 0.05 for non-normally distributed data described as median (quartiles), Mann–Whitney *U*-test. Collected count data (gender) are expressed in (%), data were unordered using ^2^ or Fisher's exact test and data were ordered using the Mann–Whitney *U* test. Consistency of image segmentation was evaluated using ICC. The area under the subject operating characteristic curve (ROC) (AUC) was used to evaluate the predictive value of the model. *P* < 0.05 was considered a statistically significant difference for comparison between groups. Other data processing was done in the deep learning algorithm program.

## Results

### Comparison of general information

There was no statistically significant difference in the comparison of general information (age, gender, BMI, systolic blood pressure, diastolic blood pressure, HR) between the three groups, *P* > 0.05 (Table 1).

**Table 1:** Comparison of general information

Indicators	Training group (*n* = 1 06 cases)	Validation group (*n* = 26 cases)	Test group (*n* = 26 cases)	*F*/*U*	*P*
Age (years)	52.16 ± 3.16	52.95 ± 3.18	53.04 ± 3.22	2.681	0.070
Gender					
Male	56	13	14	0.165	0.921
Female	50	13	12		
BMI (kg/m^2^)	21.34 ± 3.05	20.69 ± 3.06	21.62 ± 2.97	1.692	0.186
Systolic blood pressure (mmHg)	111.43 ± 12.69	111.63 ± 13.11	110.78 ± 13.26	0.083	0.920
Diastolic blood pressure (mmHg)	72.13 ± 6.32	73.12 ± 6.41	73.56 ± 5.98	1.519	0.221
HR (times/min)	70.62 ± 5.11	71.36 ± 5.62	71.59 ± 5.64	1.024	0.360

### Univariate analysis of the training group

The differences in age, gender, BMI, systolic blood pressure, diastolic blood pressure and HR between patients in the concurrent and non-concurrent groups in the training group were not statistically significant, *P* > 0.05. The rate of change in vortex area (14.10 ± 2.46)%, the rate of change in circulatory intensity (34.68 ± 5.22)%, the mean blood flow velocity (8.02 ± 2.09) cm/s, the mean EL ( 5.16 ± 0.96) J/ms, LAVI (31.27 ± 4.28) mL/m^2^, *E*/*e*' (13.21 ± 2.63) were significantly lower than the concurrent group's vortex area change rate (15.87 ± 2.51) %, circulation intensity change rate (38.39 ± 5.36) %, mean blood flow velocity (9.19 ± 2.03) cm/s, mean EL (5.99 ± 1.04) J/ms, LAVI (36.22 ± 4.15) mL/m^2^, *E*/*e*' (15.97 ± 2.87), LVEF (59.55 ± 4.66) % was significantly higher in the uncomplicated group than in the complicated group (55.43 ± 4.31) %, with statistically significant differences, *P* < 0.05 (Table 2).

**Table 2:** Univariate analysis of training groups

Indicators		Concurrent groups (*n* = 39 examples)	Non-concurrent group (*n* = 67 cases)	*F*/^2^	*P*
Age (years)		53.05 ± 3.19	52.71 ± 3.18	0.725	0.470
Gender					
Male		21	35	0.255	0.614
Female		18	32		
BMI (kg/m^2^)		21.66 ± 3.08	21.51 ± 3.09	0.330	0.742
Systolic blood pressure (mmHg)		110.29 ± 11.46	111.22 ± 13.02	0.512	0.610
Diastolic blood pressure (mmHg)		73.02 ± 6.18	72.29 ± 6.27	0.795	0.427
HR (times/min)		71.02 ± 5.33	71.66 ± 5.58	0.794	0.428
LVEF (%)		55.43 ± 4.31	59.55 ± 4.66	6.206	0.000
LAVI (mL/m^2^)		36.22 ± 4.15	31.27 ± 4.28	7.960	0.000
E/e'		15.97 ± 2.87	13.21 ± 2.63	6.835	0.000
Vortex area change rate (%)		15.87 ± 2.51	14.10 ± 2.46	3.104	0.003
Cycle strength change (%)		38.39 ± 5.36	34.68 ± 5.22	3.057	0.003
Mean blood flow velocity (cm/s)		9.19 ± 2.03	8.02 ± 2.09	2.476	0.016
Average EL (J/ms)		5.99 ± 1.04	5.16 ± 0.96	3.610	0.001

### Binary logistic regression analysis of factors influencing arrhythmias complicating hypertrophic cardiomyopathy

**Fig. 3:**
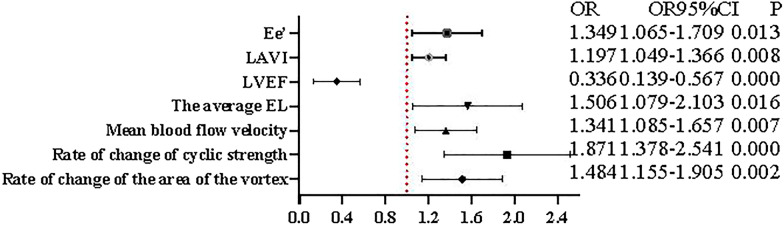
Factors influencing arrhythmias complicating hypertrophic cardiomyopathy

The factors influencing significant differences in Tab. 2 were included in a multifactorial logistic regression analysis with the dependent variable concomitant arrhythmias = 1 and non-concomitant arrhythmias = 0. The results showed that rate of change in vortex area, rate of change in circulatory intensity, mean blood flow velocity, mean EL, LAVI, and E/e' were all risk factors for concomitant arrhythmias in hypertrophic cardiomyopathy and LVEF was a protective factor for concomitant arrhythmias in hypertrophic cardiomyopathy factors. The model equation is Log(P) = rate of change of vortex area × 0.395 + rate of change of circulatory intensity × 0.627 + mean blood flow velocity × 0.293 + mean EL × 0.410-LVEF × 0.290 + LAVI × 0.180 + E/e' × 0.299–68.142 (Fig. 3).

### SE-ResNext-50's ability to recognise images

**Fig. 4:**
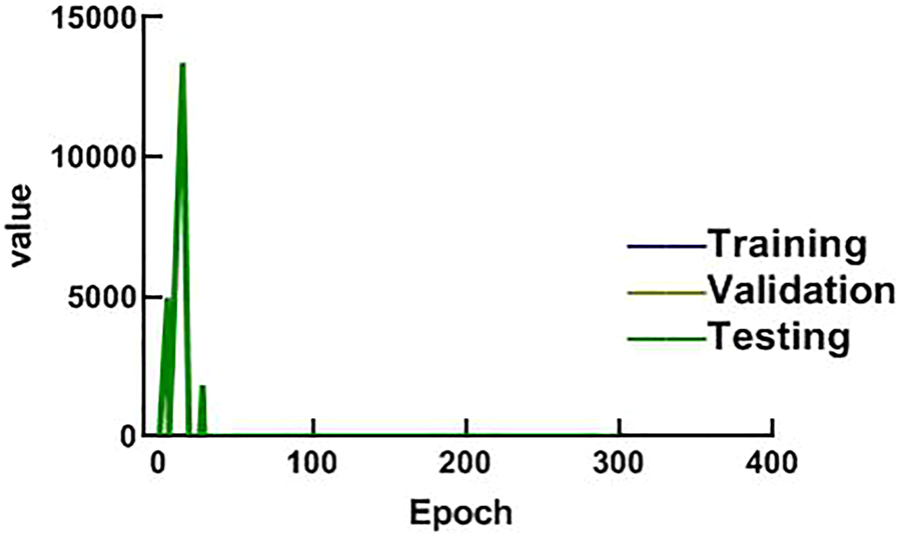
Loss curve of the model

**Fig. 5:**
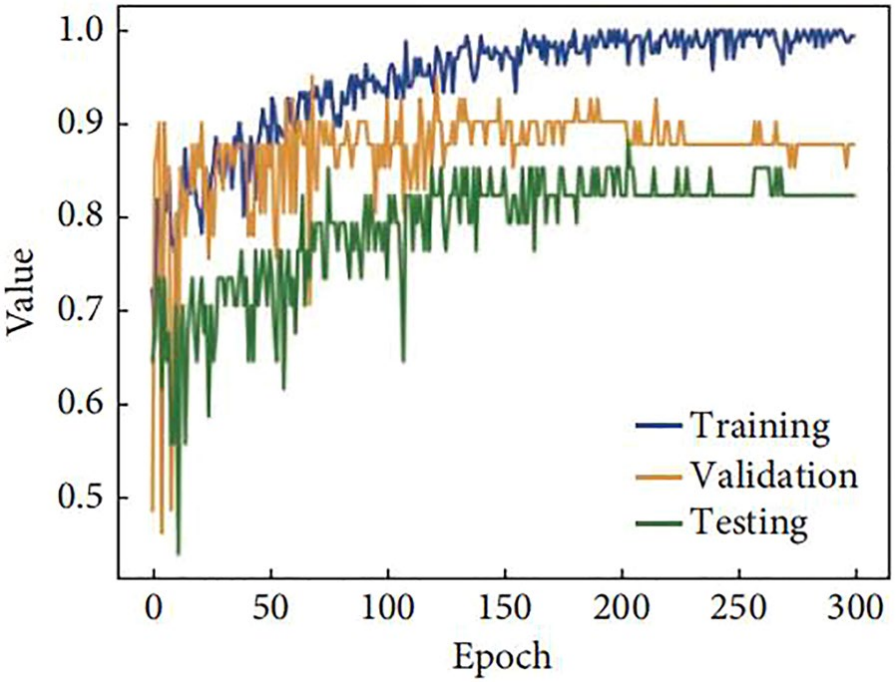
Acc curve of the model

A total of 158 images from patients were entered into the SE-ResNext-50 model. The agreement between the two physicians for the segmented area of all images was good (ICC = 0.903). The loss function Loss and acc change curves of the training showed (Figs. 4, 5) that the Loss change curve was essentially zero at epoch of 300, and the acc change curves of the training, validation and test groups gradually levelled off in value when epoch reached 300, indicating that the training did not show overfitting.

### Diagnostic efficacy of deep learning cardiac ultrasound blood flow techniques

**Fig. 6:**
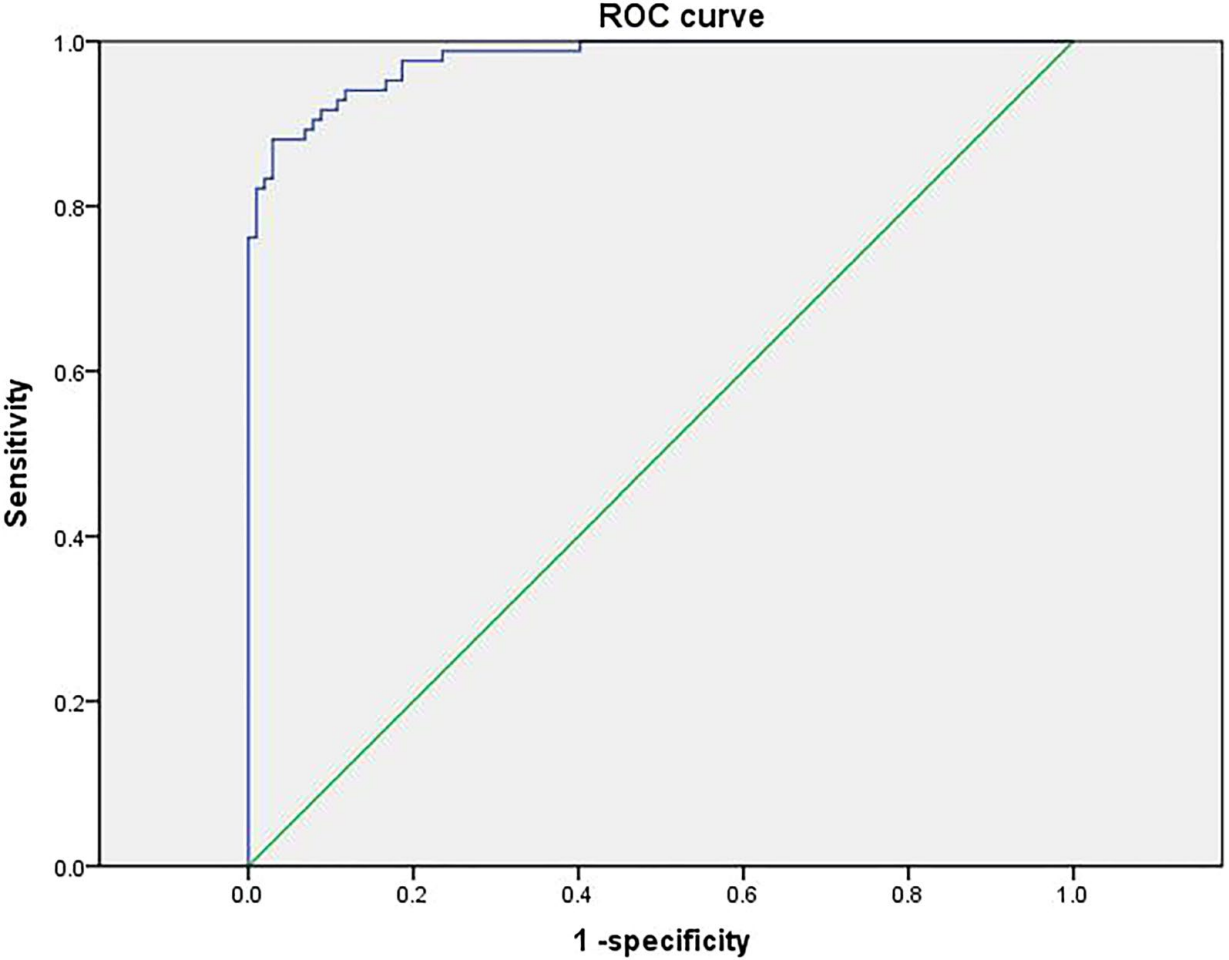
ROC curve of the training group model

**Fig. 7:**
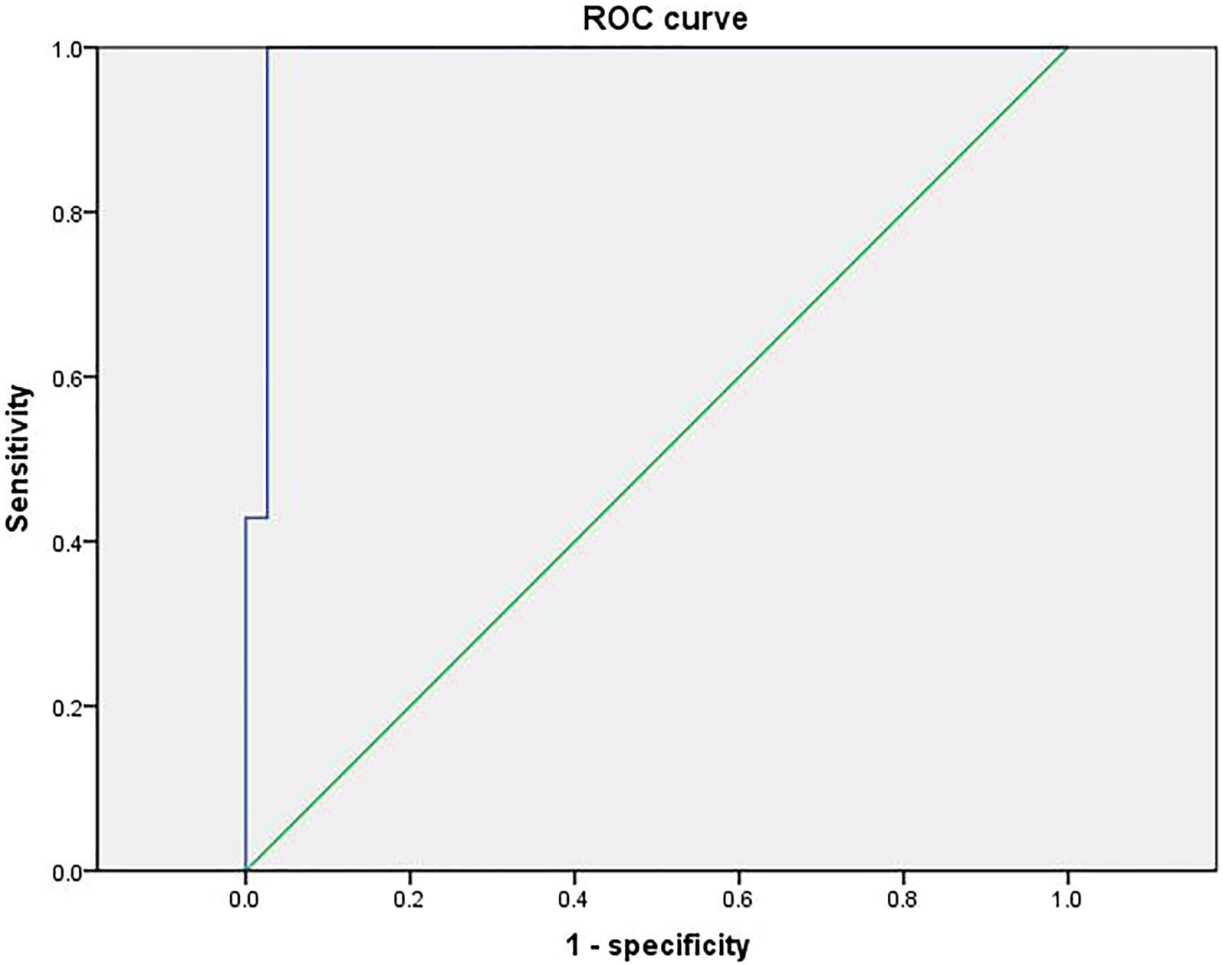
ROC curve of the validation group model

**Fig. 8:**
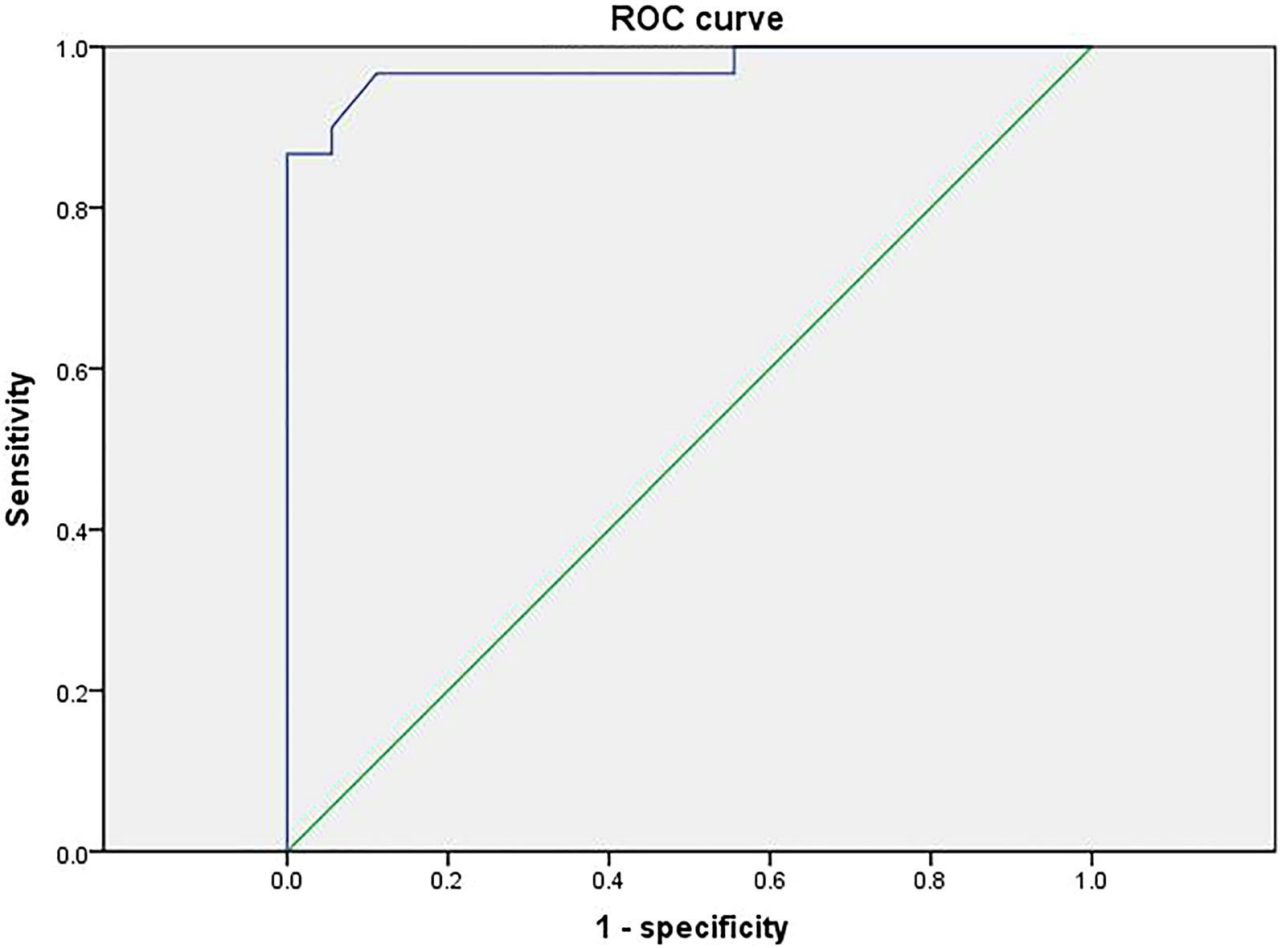
ROC curve for the test group model

The training group had a sensitivity of 0.940 and specificity of 0.882, the training group model ROC curve AUC value of 0.978, the validation group ROC curve AUC value of 0.985, sensitivity of 1.000 and specificity of 0.974, and the test group ROC curve AUC value of 0.974, sensitivity of 0.867 and specificity of 1.000 (Figs. 6, 7, 8).

## Discussion

HCM is a primary cardiomyopathy caused by structural and functional abnormalities of the myocardium, and arrhythmias are a common complication in patients with HCM [18]. Until now, the prediction of the risk of sudden death due to arrhythmias in HCM has been poorly identified [19]. The incidence of arrhythmias in patients with HCM in this study was 39.2% (62/158), which also indicates a high risk of arrhythmias in patients with HCM, and once arrhythmias occur in patients with HCM, they can lead to atrial thrombosis and ventricular tachycardia, increasing the risk of heart failure, stroke and sudden cardiac death [20]. Therefore, it is essential to analyse the risk factors for screening patients with HCM for complications of arrhythmias and their prediction. This is why it is essential to analyse the risk factors and their prediction in patients with HCM. In this study, a deep learning model was developed using cardiac ultrasound flow imaging to predict the risk of complications of arrhythmias in patients with HCM.

In this study, a total of 158 patients with hypertrophic cardiomyopathy were selected and divided into training group, validation group and test group. There was no statistically significant difference between the general information of the three groups for the follow-up experiment. The patients in the three groups were divided into concurrent and non-concurrent groups according to whether they were complicated by arrhythmias or not. The differences in age, gender, BMI, systolic blood pressure, diastolic blood pressure and HR between the patients in the concurrent and non-concurrent groups in the training group were not statistically significant, *P* > 0.05. The rate of change of vortex area, rate of change of circulatory intensity, mean blood flow velocity, mean EL, LAVI and *E*/*e*' in the non-concurrent group were significantly lower than those of the concurrent group, and LVEF was significantly higher in patients in the non-concurrent group than in the concurrent group. A multifactorial analysis later showed that vortex area rate of change, circulatory intensity rate of change, mean blood flow velocity, mean EL, LAVI, and *E*/*e*' were all risk factors for arrhythmias in hypertrophic cardiomyopathy, and LVEF was a protective factor for arrhythmias in hypertrophic cardiomyopathy.VFM is a new hydrodynamic evaluation technique for visual display and quantitative assessment. The incongruity of the luminal canal configuration and wall due to cardiomyopathy and valvular disease affects the formation and movement of vortex flow, thus increasing the level of EL. Abnormal changes in EL can lead to changes in the overall structure and function of the myocardium, so the location, morphology and extent of vortex formation are highly relevant to the structure and function of the left ventricle, and in addition patients with arrhythmias have significantly abnormal blood flow velocity indicators [21, 22]. The rate of change in vortex area, mean blood flow velocity and mean EL in patients with HCM complicated by arrhythmia were also consistent with the results of this study.

Previous studies have shown [23] that LVEF and diastolic function are protective factors against complications of arrhythmias in patients with HCM. LVEF is a quantitative indicator of cardiac systolic function, and a greater LVEF value indicates greater myocardial contractility and a lower incidence of arrhythmias. Atrial arrhythmias can be caused by increased left atrial internal diameter, increased left atrial pressure, disproportionate enlargement, atrial myocardial degeneration, increased stress, and inconsistent conduction and nonconformity. In addition, in the study by Casella et al. [24] cardiomyopathy complicated by arrhythmias, cardiac ultrasound showed an increased right atrial right ventricular internal diameter and a widened right ventricular outflow tract internal diameter, and the right atrial right ventricular internal diameter was larger than that of patients with cardiomyopathy. This also suggests that the right atrial intraventricular diameter may be a factor in arrhythmias associated with cardiomyopathy. Significantly elevated LAVI has been found in patients with arrhythmias and its role in the development of arrhythmias [25]. The E/e' ratio has been widely used in the assessment of left ventricular diastolic filling pressures in various cardiac disease processes. Impaired left ventricular diastolic function and the resulting increase in left ventricular filling pressure can lead to stagnation of blood flow in the left atrium and left atrial thrombosis [26]. Therefore, in addition to indicating increased LV diastolic filling pressures, an increase in the *E*/*e*' ratio may also indicate an increased risk of left atrial stasis and thrombosis, which can be a cause of sudden cardiac death in arrhythmias. Finally the loss function Loss and acc change curves for the deep learning model training established in this study showed that the training for did not show overfitting, indicating a better model. In addition, the ROC curve AUC value for the model in the training group was 0.978, the ROC curve AUC value for the validation group was 0.985 and the ROC curve AUC value for the test group was 0.974, with high sensitivity and specificity and high predictive value.

In summary, deep learning-based cardiac ultrasound flow imaging can identify cardiac ultrasound images more accurately and has a high predictive value for arrhythmias complicating hypertrophic cardiomyopathy, and vortex area change rate, circulation intensity change rate, mean flow velocity, mean EL, LAVI, and *E*/*e*' are all risk factors for arrhythmias complicating hypertrophic cardiomyopathy, and LVEF is a Protective factors for arrhythmias in hypertrophic cardiomyopathy. The use of cardiac ultrasound flow imaging should focus on abnormalities in these parameters to avoid arrhythmias in hypertrophic cardiomyopathy. There are some limitations to this study. Due to the limited sample size of this study, future studies should include a larger sample size to explore the effectiveness of deep learning models to identify arrhythmias complicating hypertrophic cardiomyopathy. In addition, cardiac ultrasound flow imaging was used in this study, and subsequent studies could use combined diagnostic images for deep learning modelling to better predict arrhythmias complicating hypertrophic cardiomyopathy.

## Data Availability

The datasets used and/or analysed during the current study are available from the corresponding author on reasonable request.
